# Supplementary material for: Hydrocarbon-Stapled Peptide Based-Nanoparticles for siRNA Delivery
Source: Nanomaterials (Basel). 2020 Nov 25;10(12):2334. doi: 10.3390/nano10122334 (PMC7760004; doi:10.3390/nano10122334)
Supplement: Supplementary file 1 [file nanomaterials-10-02334-s001.pdf]

nanomaterials

Supplementary Material

Hydrocarbon-stapled peptide based-nanoparticles for siRNA delivery

Matthieu Simon[1,2,†], Nabila Laroui [1,†], Marianne Heyraud [1], Guillaume Laconde [1], Lamiaa M.A. Ali [1,3], Kevin Bourbiaux [1], Gilles Subra [1], Lubomir L. Vezenkov [1], Baptiste Legrand [1], Muriel Amblard [1,*], Nadir Bettache [1,*]

1 IBMM, Univ Montpellier, CNRS, ENSCM, 34093, Montpellier, France. matthieusimon.work@gmail.com (M.S.); nabila.laroui@etu.umontpellier.fr (N.L.); marianneheyraud@hotmail.fr (M.H.); guillaumelaconde@gmail.com (G.L.); lamiaa.ali@umontpellier.fr (L.M.A.A.); kevin.bourbiaux@gmail.com (K.B.); gilles.subra@umontpellier.fr (G.S.); lubomir.vezenkov@enscm.fr (L.L.V.); baptiste.legrand@umontpellier.fr (B.L.); muriel.amblard@umontpellier.fr (M.A.); nadir.bettache@umontpellier.fr (N.B.)

[2] Present address: IRMB, CARTIGEN, CHU de Montpellier, 34090 Montpellier, France. matthieusimon.work@gmail.com (M.S.)

[3] Department of Biochemistry, Medical Research Institute, University of Alexandria, Alexandria 21561, Egypt. lamiaa.ali@umontpellier.fr (L.M.A.A.)

* Correspondence: muriel.amblard@umontpellier.fr (M.A.); nadir.bettache@umontpellier.fr (N.B.)

[†] These authors contributed equally to this work.

1. Materials and Methods 2

2. Results 2

2.1. Cytotoxicity study of stapled peptides in reducing conditions 2

2.2. Gel electrophoresis of the complex JMV6337/siRNA 3

2.3. Luciferase activity of JMV6337 on MDA-MB-231-Luc-RFP cells 3

2.4. Cellular uptake quantified by flow cytometric analysis of MDA-MB-231 cells 4

2.5. Luciferase activity using siCtrl complexed with stapled peptides in MDA-MB-231-Luc-RFP cells 4

Supplementary Material

1. *Materials and Methods*

Internalization Quantification by Flow Cytometry

Cells were treated or not with Stapled peptides/siCtrl-cy5 (100 nM) at N/P = 5 for JMV6582 and N/P = 2 for JMV6580 and JMV6583 for 4 h. After treatment, control and treated cells were washed once in cold Phosphate Buffer Saline (PBS), harvested and centrifuged (1300 rpm, 5 min). Cell pellet were resuspended in PBS enriched with $CaCl_2$ + $MgCl_2$+ and stained with propidium iodide (1 µg.mL^{-1}) (Sigma-Aldrich Chimie, Lyon, France), a cell death indicator. Flow cytometric determination of living cells and treated positive cells was done by FACS Novocyte Flow Cytometer (ACEA Biosciences, Inc., San Diego, CA, USA) with a minimum of 20,000 living cells collected. Data were analysed with NovoExpress software (ACEA Biosciences, Inc., San Diego, CA, USA).

2. *Results*

2.1. Cytotoxicity study of stapled peptides in reducing conditions

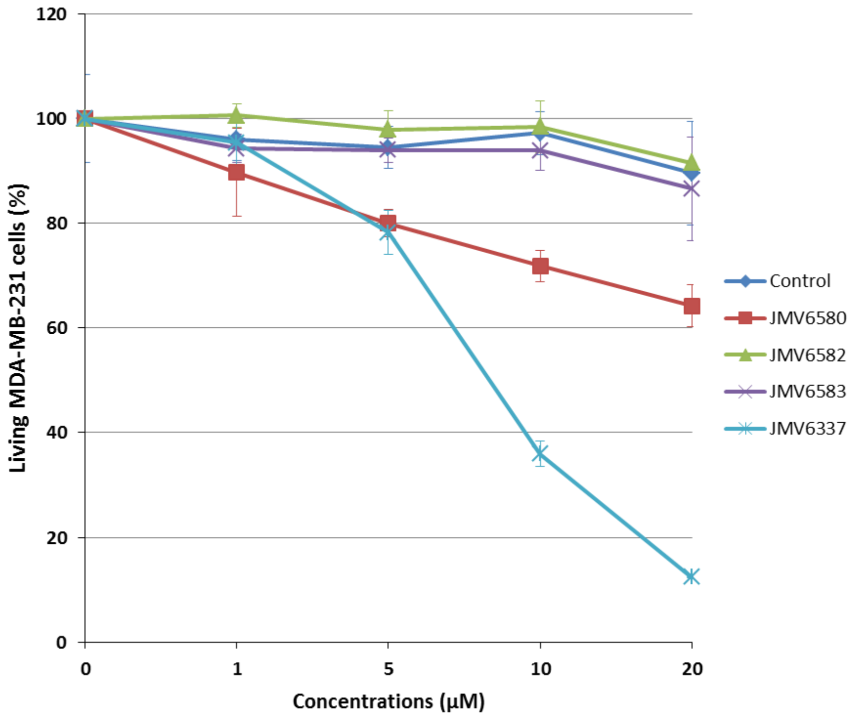

Figure S1 : Cytotoxicity study; human breast adenocarcinoma MDA-MB-231 cells were incubated with increasing concentrations (from 0 to 20 µM) of the stapled peptides and an excess of 10 equivalents of DTT (from 0 to 200 µM), respectively, for 72 h. Results are presented as means ± standard deviations of three independent experiments performed in triplicate.

2.2. Gel electrophoresis of the complex JMV6337/siRNA

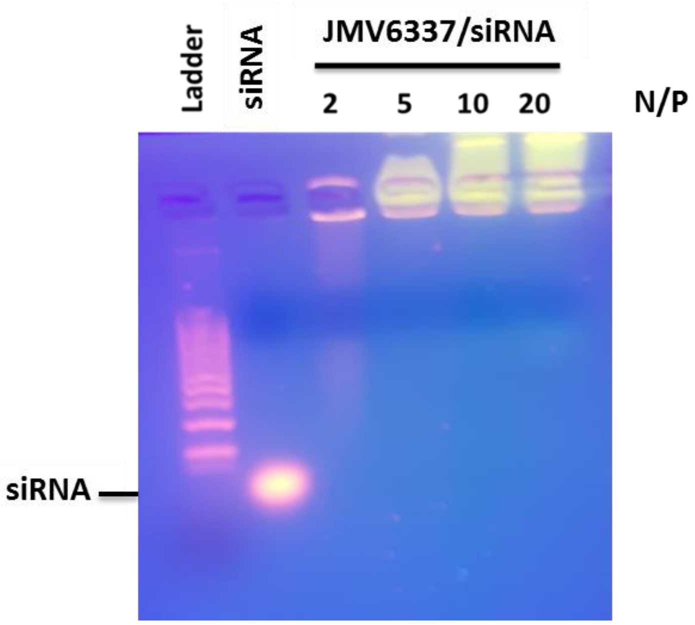

Figure S2: JMV6337 stapled peptide complexation with siRNA monitored by agarose gel electrophoresis analysis at N/P = 2, 5, 10 and 20.

2.3. Luciferase activity of JMV6337 on MDA-MB-231-Luc-RFP cells

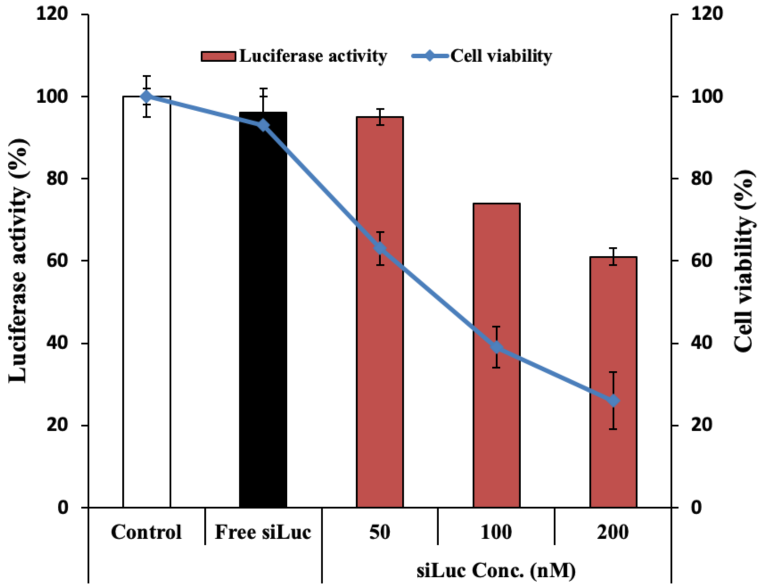

Figure S3: Luciferase activity assay showing the transfection of a 21-mer siRNA targeting the expression of luciferase inside MDA-MB-231-Luc-RFP cells. The experiments were carried out with increasing amounts of siLuc (from 50 to 200 nM) complexed with the JMV6337 stapled peptide at N/P = 2. The corresponding concentrations of the JMV6337 are from 1.05 μM to 4.2 μM.

2.4. Cellular uptake quantified by flow cytometric analysis of MDA-MB-231 cells

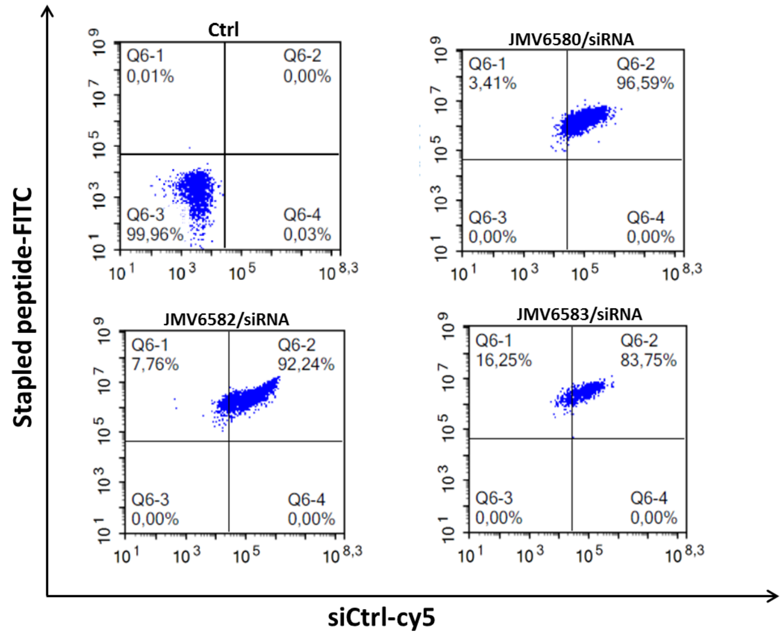

Figure S4: Cellular uptake quantified by flow cytometric analysis of MDA-MB-231 cells treated with stapled peptide/siRNA complexes. MDA-MB 231 cells alone (upper left), cells incubated with the complex formed between JMV6580 and siCtrl-cy5 at N/P = 2(upper right), cells incubated the complex formed between JMV6582 and siCtrl-cy5 at N/P = 5 (lower left) and finally, cells incubated with the complex formed between JMV6583 and siCtrl-cy5 at N/P = 2 (lower right). Numbers in the profiles indicate the percentage of cells present in this area.

2.5. Luciferase activity using siCtrl complexed with stapled peptides in MDA-MB-231-Luc-RFP cells

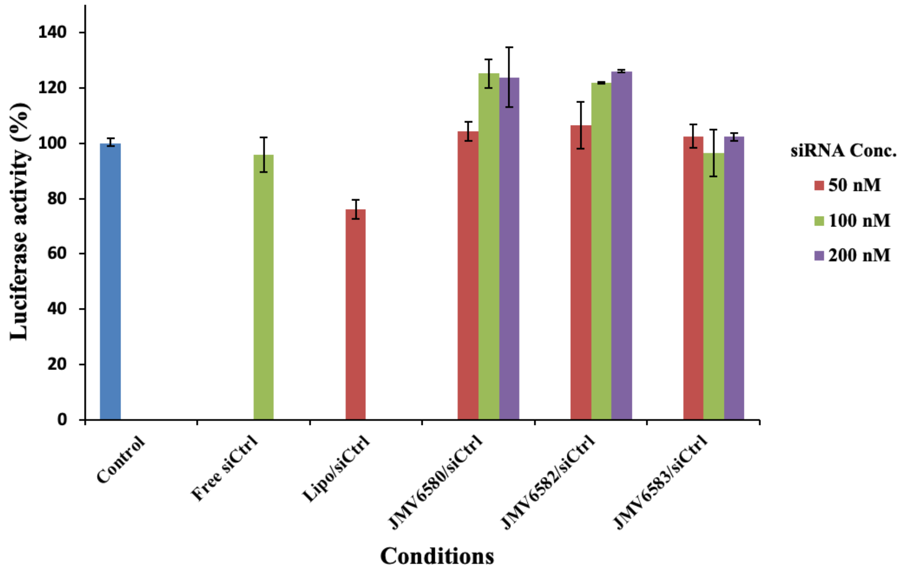

Figure S5: Luciferase activity assay showing the transfection of a non-targeting 21-mer siRNA inside MDA-MB-231-Luc-RFP cells. The experiments were carried out with increasing amounts of siCtrl (from 50 to 200 nM) complexed with the stapled peptides. The corresponding concentrations of the peptides are from 1.05 μM to 4.2

μM for JMV6580, from 1.4 μM to 5.6 μM for JMV6583 and from 5.25 μM to 21 μM for JMV6582. The concentration of the siCtrl used in Lipofectamine condition is 50 nM.
